# Longitudinal Language-Model Reasoning Enables Automated Labeling of Lung Cancer Recurrence from Unstructured Clinical Records

**DOI:** 10.21203/rs.3.rs-9550278/v1

**Published:** 2026-05-21

**Authors:** Carlotta S Hoelzle, Johannes Brandt, Jonathan C. Mueller, Maximiliano Klug, Julian Westphal, Daniel Rueckert, Maulik Chevli, Florian J. Fintelmann

**Affiliations:** 1* Department of Radiology, Massachusetts General Hospital, Boston, United States of America.; 2 Chair of AI in Healthcare and Medicine, Technical University of Munich, Munich, Germany.; 3 Department of Computing, Imperial College London, London, UK.; 4 Munich Center for Machine Learning (MCML), Munich, Germany.

**Keywords:** Large Language Models, Endpoint Extraction, Electronic Health Record, Real World Evidence

## Abstract

Many clinical endpoints are rarely captured as structured variables, necessitating labor-intensive manual abstraction from longitudinal narratives. We present SCRIBE, an open-source, training-free framework that extracts temporally precise, auditable clinical labels from unstructured records using only narrative text. SCRIBE utilizes multi-stage large language model reasoning to reconcile longitudinal evidence into accurate event labels and their timing while retaining verbatim evidence linked to original source records. This traceability enables efficient expert verification and streamlines radiologic review by pinpointing exact diagnostic windows. In a multi-center cohort of 2,065 patient’s with lung cancer, SCRIBE achieved high recurrence detection performance, halved temporal localization error compared to note-level inference and reduced total token volume of multi-year patient documentation by nearly two orders of magnitude. Notably, expert adjudication revealed that 47.8% of false positives were valid events missing from official registries. These results demonstrate SCRIBE’s capacity to automate high-fidelity endpoint extraction while auditing and improving the completeness of real-world clinical registries.

## 1 Introduction

Cancer recurrence, treatment failure, and other clinically consequential outcomes are rarely stored as a single structured variable in electronic health records (EHRs) [1, 2]. Instead, they must be inferred from evidence distributed across longitudinal clinical documentation, including radiology reports, pathology findings, operative notes, and serial physician assessments spanning months to years [3, 4]. Manual abstraction remains the gold standard for generating temporally precise patient-labels [5, 6]. However, this process is prohibitively labor-intensive and fails to scale to the cohort sizes required for modern machine learning. Critically, definitive confirmation of oncological endpoints often requires expert image interpretation; yet, there is a lack of tools to efficiently pinpoint specific evidence or temporal windows, which would allow experts to verify predictions against imaging without reviewing the entire patient history. Structured alternatives such as administrative billing codes and institutional disease registries offer scalability, but they provide an incomplete and temporally coarse representation of clinical events [7]. These systems typically depend on manual abstraction workflows, capture only confirmed outcomes, and often omit the diagnostic trajectory that precedes formal documentation [2, 8]. For endpoints such as lung cancer recurrence this can lead to under-ascertainment and loss of temporal fidelity.

Automatic extraction of definitive evidence from unstructured text contained in the EHR to resolve clinical endpoints remains challenging. Rule-based systems and regular expressions (REGEX) are brittle to linguistic and institutional variation, and cannot reconcile conflicting signals across multi-year patient histories [9, 10]. Supervised clinical text classifiers such as ClinicalBERT and BioBERT improve local information extraction from individual notes [11–13], but their training relies on large, annotated datasets which need to be manual constructed and reviewed. Recent advances in general large language models (LLMs) offer a more flexible alternative for clinical text reasoning [14–19], yet current applications remain limited. Current models often lack grounding in verifiable source text, limiting auditability in high-stakes clinical environments [20, 21]. Moreover, existing approaches typically process documents in isolation [14] or restrict analysis to a single modality [16, 22], failing to cover the complete patient history. This issue is accompanied by inherent constraints in model context windows, frequently necessitate extensive text preprocessing or the outright exclusion of lengthy clinical records, potentially discarding important clinical records required for accurate assessment [9, 16, 23, 24]. Furthermore, many high-performing architectures depend on substantial computational resources or use closed-source models, restricting deployment within institutional firewalls and reducing scalability and reproducibility across healthcare systems [14, 23, 25, 26].

To unlock the potential of longitudinal electronic health records, an automated framework must resolve three intertwined challenges: (1) extracting verifiable evidence from unstructured notes; (2) compressing multi-year documentation into computationally manageable formats; and (3) applying global reasoning to ensure temporal consistency.

To address this, we present SCRIBE (Scalable Clinical Reasoning for Information and Biomarker Extraction), a training-free framework for deriving structured, time-resolved clinical labels from longitudinal EHR narratives. SCRIBE operates as a multi-stage reasoning pipeline that reflects the sequential nature of clinical decisionmaking over a patient’s full text-based clinical history. In the first stage, a lightweight, on-premise LLM processes individual clinical notes to identify candidate events, extracting both structured labels and verbatim textual evidence. These note-level signals are then consolidated through temporal aggregation into a continuous, month-resolved patient timeline. In the final stage, the same LLM performs global reasoning over the aggregated history to reconcile conflicting signals, enforce temporal consistency, and produce a coherent patient-level interpretation of the clinical trajectory. By anchoring every prediction to specific notes and verbatim evidence chains, the framework provides a transparent audit trail. This allows clinicians and researchers to trace a predicted endpoint back to the exact source record, facilitating rapid verification of imaging and enabling the construction of high-fidelity research datasets.

We validate SCRIBE on lung cancer recurrence as a prototypical longitudinal endpoint, using a retrospective multi-center cohort of 2,065 patients across eight hospitals. Our results demonstrate that global reasoning significantly improves temporal localization by pinpointing exact event dates and reconciles cross-document contradictions. Furthermore, our robustness analysis confirms that while note-level extraction is sensitive to prompting, the global reasoning stage produces stable, near-deterministic outputs. By revealing valid clinical endpoints absent from institutional registries, SCRIBE demonstrates the capacity to automate high-fidelity endpoint extraction while providing the transparency required for clinical and radiological review.

## 2 Results

### 2.1 Study design and data source

The SCRIBE pipeline (Figure 4) facilitates automated lung cancer recurrence labeling from longitudinal EHRs. For each month in which clinical documentation is available, the pipeline assigns one of three event labels, specifically recurrence (REC), a suspicious event (SUSP), or no evidence of recurrence (NOREC), alongside the extracted supporting evidence and the model’s reasoning. For months classified as REC, the pipeline additionally outputs the anatomical location of the recurrence. This framework was evaluated on a registry-derived cohort of 2,065 patients across eight hospitals, with an ablation subset (GT2; n = 398) utilized for systematic experimentation. Analysis focused on two unstructured-text modalities, ambulatory visit summaries (VIS) and inpatient progress reports (PRG). Given its slight superior performance, PRG served as the primary input for ablation studies. System performance was quantified via **P**atient-level **R**ecurrence **D**etection (PRD) and **T**emporal **E**vent **L**ocalization (TEL). While PRD validates the correct attribution of recurrence to a patient, TEL measures the precision of the predicted date within ±1, ±2, and ±3-month windows alongside the Mean Absolute Error (MAE). All inference occurred within the institutional firewall without model fine-tuning or exposure to registry labels.

### 2.2 SCRIBE performance

SCRIBE employs a two-stage architecture designed to synthesize the full patient history while mitigating the ”lost in the middle” effect and context window constraints typical of long clinical histories. First, SCRIBE executes note-level extraction (NLE) to capture granular evidence, followed by a global reasoning (GR) step that adjudicates these signals across the entire longitudinal timeline. The transition to global reasoning maintained stable PRD while substantially enhancing TEL. For PRG, longitudinal adjudication increased PRD F1-scores to 72.9% and TEL F1-scores to 80.3%. Similar improvements were observed using VIS notes as input.

The primary impact of global reasoning was a significant reduction in temporal error and a sharp increase in localization reliability. Most notably, the F1-score for PRG-based TEL increased by more than 10 percentage points (from 69.8% to 80.3%), with significant F1 improvements also observed for joint integration of VIS and PRG as input modalities. For all significance tests refer to Supplementary Table C5. Across all modalities, MAE decreased and within-one-month accuracy improved significantly (Wilcoxon signed-rank *P* < 0.001; Bonferroni-adjusted *α* = 0.025), with the latter exceeding 86% for all input modality variation. Following GR, precision increased and confidence intervals narrowed substantially while recall remained high. We report the full performance details in Table 1. This indicates that the global reasoning step effectively filters local inconsistencies by contextualizing them within the entire clinical history. Performance remained robust in the full cohort, which is 4.5-fold larger than GT2, where PRD F1 reached 73.0%, TEL F1 80.6% and MAE was reduced from 1.30 to 0.67 months.

Comparison against our deterministic regex baseline confirmed that surface-level pattern matching is insufficient for this task, as it yielded near-absent specificity (13.5%) and high temporal error (MAE 1.73 months), see Supplementary Table C4.

### 2.3 Cross-modality consistency and rationale for PRG-based ablations

To assess modality dependence, we compared global reasoning outputs derived from VIS, PRG, and combined VIS+PRG inputs using paired McNemar tests for PRD and TEL and paired Wilcoxon signed-rank tests for temporal error. PRD performance did not significantly differ between PRG and VIS (*P* = 0.79). TEL F1 was significantly more robust using PRG versus VIS (*P* = 0.00008), reflecting a higher reliability of progress reports for event dating. This advantage was driven primarily by higher precision at similar recall, whereas temporal error did not differ significantly between the two modalities (*P* = 0.79). All results are shown in Table 2.

**Fig. 1:**
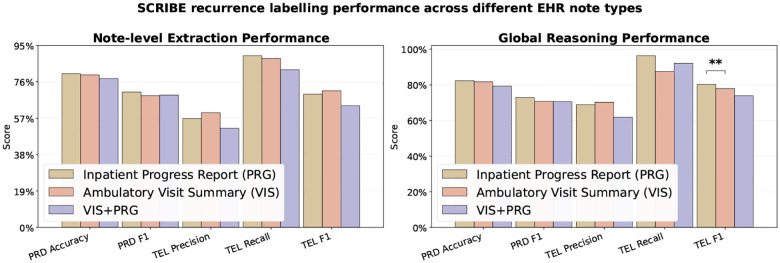
SCRIBE performance across modalities and pipeline stages. Performance of the SCRIBE pipeline across documentation modalities and processing stages. (a) Note-level extraction performance and (b) global reasoning performance are shown for inpatient progress reports (PRG), ambulatory visit summaries (VIS), and their combination (VIS+PRG). Metrics include patient-level recurrence detection (PRD; accuracy and F1) and temporal event localization (TEL; precision, recall, and F1). Global reasoning improves temporal localization performance across all modalities, with the largest gains observed in TEL recall. Differences between modalities are modest, indicating that a single high-coverage source captures most clinically relevant signal. Paired McNemar tests were used for selected comparisons; ** indicates *P* < 0.017 (Bonferri adjusted).

The integration of VIS and PRG modalities yielded no significant performance gain over the use of PRG alone across PRD, TEL, or MAE metrics (Table 1). While the combined pipeline showed a nominal TEL improvement of PRG over VIS, this effect did not remain significant after Bonferroni correction and was not accompanied by a significant reduction in MAE. Given that PRG independently produced the most robust global TEL profile, achieving peak F1 and precision while maintaining high recall (Fig. 1), it was selected as the primary input modality for all subsequent ablation experiments.

### 2.4 System robustness and stability

Analysis of prompting strategies revealed a distinct divergence in architectural sensitivity between the two pipeline stages. Note-level extraction exhibited high sensitivity to prompt design across eight structured strategies, including Chain-of-Thought (CoT), Tree-of-Thought (ToT), and schema-optimized variants. Performance varied by up to 13% in PRD and TEL F1 compared to the manual constructed clinician-informed base prompt, with most deviations reaching statistical significance (McNemar *P* < 0.001 after Bonferroni correction; Supplementary Tables C10 and C11). In particular, modifying the output JSON schema from binary evidence fields per label to one categorical representation led to consistent performance degradation, highlighting the criticality of structured output design for evidence extraction.

Conversely, the global reasoning stage demonstrated marked robustness. No statistically significant differences were observed across prompting configurations (*P* > 0.05), with minimal variation in F1 (<5%) and overlapping confidence intervals; Supplementary Tables C13 and C12. These results suggest that longitudinal reasoning is comparatively insensitive to prompt architecture once high-fidelity evidence is established.

Further evaluation of inference stability through run-repeatability and temperature-sensitivity analyses (0.1, 0.7, 1.0) confirmed system reliability. Wilcoxon singed-rank tests indicated that neither sampling temperatures nor repetitions of the same configuration significantly alter temporal precision (*P* > 0.0167 for all MAE comparisons). Paired McNemar tests detected no significant shifts in classification decisions across independent runs following Bonferroni correction (*α*_*adjusted*_ = 0.0167). However, McNemar tests on TEL F1 scores yielded significant differences between temperature regimes (1.0 vs. 0.1 and 0.7 vs. 0.1). When paired with the superior recall observed at lower temperatures (0.7 and 0.1) relative to 1.0, these data suggest that elevated model stochasticity, while increasing creativity, is disadvantageous to performance in this particular reasoning task. Comprehensive performance metrics and significance testing for these stability analyses are detailed in Supplementary Tables C8 through C6.

### 2.5 Expert adjudication and error analysis

#### False positive evaluation

All false positive (fp) predictions from the VIS and PRG base reference runs, yielding 134 cases out of 29,788 total predictions, were subjected to a structured post-hoc error analysis. We stratified three conceptual groups: model failure, study limitations, and registry missclassification. For a detailed breakup of categories see Table 3.

38 cases were confirmed as primary model errors, the remaining 96 cases were escalated to a board-certified thoracic radiologist for review of all available imaging studies (MK). Manual error analysis revealed that strict semantic hallucination occurred in 2 cases (1.4%), in which the model invented a confirmed recurrence based on suspicious findings in the radiology report. A conceptually distinct form of fabrication, compositional hallucination through temporal reasoning drift, in which a correctly identified event was anchored to an unrelated note date, was identified in 8 cases (5.6%). Of the 38 model failures, temporal grounding errors were the primary cause (21.6%), stemming from misalignments between clinical events and their attributed timestamps. Analysis of the 96 clinically reviewed cases revealed that 32 were classified as false positives due to study-level limitations despite accurate model reasoning. These cases were primarily driven by domain knowledge boundaries (17 cases) and duplicate ascertainment (12 cases), highlighting constraints inherent to prompt design and institutional recording practices.

Expert adjudication identified substantial registry under-ascertainment. 47.8% of initially classified false positives were reclassified as correct model predictions.

**Fig. 2:**
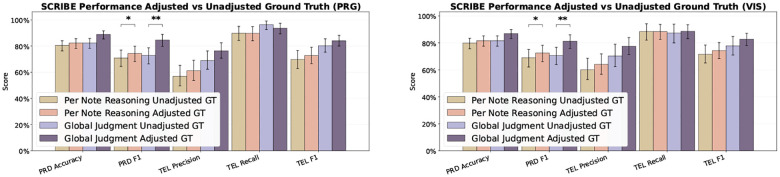
SCRIBE performance with adjusted versus unadjusted ground truth labels across pipeline stages and documentation modalities. Impact of expert-corrected ground truth labels on SCRIBE performance across pipeline stages. (a) Inpatient progress reports (PRG) and (b) ambulatory visit summaries (VIS) are shown for both note-level extraction and global reasoning stages, evaluated against registry-derived (unadjusted) and clinician-adjudicated (adjusted) ground truth. Metrics include PRD accuracy and F1, and TEL precision, recall, and F1. Ground truth correction leads to consistent improvements across all metrics, with the largest gains observed in temporal localization (TEL), reflecting correction of registry under-ascertainment. Error bars represent 95% confidence intervals estimated via patient-level bootstrap resampling (B = 10,000). Statistical significance between adjusted and unadjusted comparisons is indicated (*P < 0.05, * * *P* < 0.025 (Bonferroni adjusted); paired McNemar tests).

Re-evaluation against the updated reference standard resulted in significant performance gains across modalities, see Figure 2. Global reasoning PRD F1 increased from 70.7% to 81.3% for VIS and from 72.9% to 84.6% for PRG (both *P* < 0.001). TEL F1 increased from 77.9% to 82.8% (VIS) and from 80.3% to 84.1% (PRG), while MAE decreased for VIS (0.57 to 0.50 months; *P* = 0.001) and remained stable for PRG.

#### False negative evaluation

False-negative predictions were manually reviewed by a computer scientist and a trained analyst. A total of 39 cases were identified, comprising 9 PRG and 30 VIS instances (Table 4).

False negatives were distributed across study limitations and model errors. In PRG, cases were evenly split between no available documentation, strict instruction boundary, and model error (*n* = 3 each). In VIS, false negatives were primarily driven by missing documentation (*n* = 13) and model errors (*n* = 12), with fewer cases due to strict instruction boundaries (*n* = 3) and design choices (*n* = 2).

Instruction boundary cases involved suspicious imaging findings without confirmatory actions, insufficient for recurrence classification under predefined criteria. Model errors occurred despite available evidence and included failures in information extraction or temporal reasoning. Cases with no available documentation were most frequent in VIS and reflected the absence of longitudinal context, with confirmatory information often limited to operative reports, which are not included in ambulatory visit summaries. Design choice cases reflected instances in which multiple recurrence signals were clustered, leading to assignment of the recurrence date to an earlier time point preceding the registry-confirmed event.

Across subcategories, the model assigned a SUSP label in 56.4% of all false-negative cases. This indicates that, in some instances, the model identified potentially relevant findings but did not meet the threshold for confirmed recurrence classification.

### 2.6 Demographic bias evaluation

Performance was broadly consistent across demographic strata, with stable PRD sensitivity (90.9–92.2%) and TEL F1-scores (74.5–80.5%) across sex and age groups, and no directional differences between male and female patients. Reduced precision in the youngest subgroup (age 30–55; n = 13) was attributable to limited sample size rather than a systematic model effect.

Race-stratified analyses were dominated by the White (Caucasian) majority (n = 363), which largely determined aggregate performance. Although minority subgroups showed numerically comparable results, statistical power was insufficient to detect clinically meaningful disparities (0.09–0.13 for a 10 pp TEL F1 difference). Accordingly, observed performance stability reflects cohort composition rather than confirmed equity across populations. We report the full subgroup performance evaluation in Supplementary Table C17.

### 2.7 Effect of documentation volume on performance

**Fig. 3:**
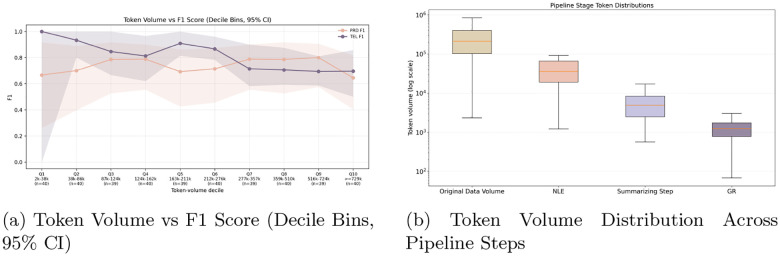
Token volume analysis and compression analysis. Relationship between documentation density, model performance, and token compression across the SCRIBE pipeline. (a) PRD F1 and TEL F1 scores are shown across ten equal-sized token-volume deciles for the global reasoning stage on the GT2 PRG cohort. Shaded regions represent 95% confidence intervals estimated via patient-level bootstrap resampling (B = 10, 000). Performance is most variable in the smallest token-volume decile, stabilizes across intermediate deciles, and PRD F1 shows modest decline at the highest volumes, reflecting increased ambiguity in highly dense documentation. (b) Token volume distributions (log scale) across pipeline stages demonstrate progressive compression from raw clinical notes to structured evidence extraction, summarization, and global reasoning inputs. Boxplots indicate median and interquartile range, illustrating substantial reduction in token volume while preserving predictive performance.

**Fig. 4:**
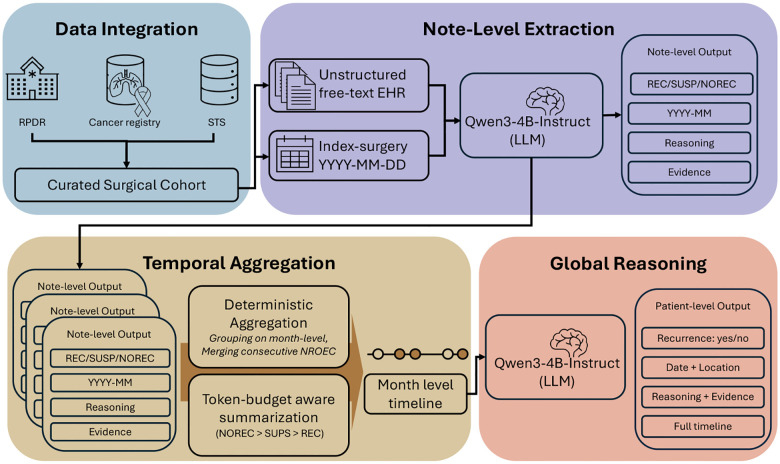
Overview of the SCRIBE pipeline for longitudinal lung-cancer recurrence extraction from electronic health records. (1) data integration and cohort construction through linkage between cancer registry and hospital data management; (2) note-level extraction using a LLM to classify clinical notes into recurrence-related categories and extract structured evidence; (3) temporal aggregation, consolidating note-level predictions into a month-level patient timeline using deterministic rules and token-budget-aware summarization; (4) global reasoning, LLM performs longitudinal consistency checking and infers recurrence outcomes, including timing and location. This hierarchical design enables scalable processing of long, unstructured clinical histories while preserving interpretability through explicit evidence extraction.

To enable longitudinal reasoning, the pipeline must compress vast clinical histories into a digestible context window without losing diagnostic fidelity. SCRIBE achieves this through a multi-stage reduction process, yielding an overall compression of approximately two orders of magnitude (Figure 3b). Median documentation volume decreased from 211,000 raw tokens to 35,000 following note-level extraction, eventually reaching approximately 1,300 tokens after global reasoning - representing a cumulative 99.4% reduction, without the need of excluding documents or other extensive preprocessing.

Analysis across ten token-volume deciles (Figure 3a) demonstrates that this compression maintains consistent performance regardless of initial documentation density. While the lowest decile (Q1) showed the highest variance, PRD F1 remained stable across mid-to-high deciles (0.70–0.80). TEL F1 exhibited a gradual decline from its Q1 peak before plateauing around 0.70 in the highest deciles (Q7–Q10). Crucially, no performance collapse or increase occurred at extreme volumes (≥729k tokens), suggesting that increased documentation volume does not necessarily improve accuracy and that SCRIBE successfully mitigates noise while preserving essential signals across the full patient history.

## 3 Discussion

We demonstrate that an open-access, zero-shot, on-premise LLM pipeline can reliably attribute lung cancer recurrence events from unstructured EHRs per patient with high sensitivity (95.3%) and specificity (85.9%). By archiving high temporal event localization (F1 of 0.84) without model training or access to structured labels, this work proves that clinical endpoints, including their temporal context and evidentiary basis, can be reconstructed directly from large-scale narrative data. This capability effectively bypasses a primary bottleneck in real-world oncology research by transforming raw clinical text into actionable datasets.

SCRIBE is the central contribution of this work. Our multi-stage architecture addresses the core challenges of evidence verification, multi-year documentation, and temporal consistency. By retaining verbatim evidence linked to the original files, SCRIBE streamlines the review process, ensuring transparency and auditability while allowing clinicians to focus on relevant temporal windows without navigating entire patient histories.

A key component of this architecture is the global reasoning stage, which cannot be treated as an auxiliary step but represents a critical determinant of overall performance. By integrating distributed evidence into a coherent longitudinal trajectory, this stage significantly improves temporal event localization and leads to consistent gains in patient-level detection performance. These findings indicate that accurate recurrence ascertainment depends primarily on cross-document aggregation and temporal adjudication rather than isolated evidence extraction. Furthermore, the global reasoning stage integrates signals into a consistent trajectory and reduces the mean absolute date error to approximately 0.5 months compared to note-level classification. This improvement suggests that recurrence identification is constrained by the ability to reconcile longitudinal evidence rather than detect individual signals, thereby enabling the automated generation of temporally precise clinical endpoint labels. Importantly, these benefits are preserved when scaling to substantially larger cohorts, indicating that the proposed architecture maintains performance under realistic data volumes and supports its applicability to large-scale clinical datasets.

Importantly, we observe that note-level evidence extraction and reasoning exhibit sensitivity to prompting strategies. Performance was highest when using a manually iterated prompt that enforces structured outputs with binary labels per category, rather than one multi-class label per note. Alternative prompting approaches were generally less effective, with the exception of CoT formulations, which achieved comparable performance when grounded in the structural reasoning framework derived from the radiologist workflow. We find that once evidence has been correctly distilled at the note level, downstream temporal adjudication during the global reasoning stage is robust to prompt variations. Following the global reasoning stage, SCRIBE produced quasi-deterministically reproducible outputs across runs, and low temperature settings, and input modalities, satisfying a critical requirement for research and clinical use.

A key finding of this study is the identification of significant underascertainment in the institutional cancer registry. Post hoc expert adjudication of 134 initial false-positive predictions revealed that 47.8% corresponded to clinically valid recurrence events not captured in the manually maintained registry. Updating the reference standard led to statistically significant improvements in F1 scores (up to Δ*F*1_*TEL*_ 5%, *P* < 0.001), highlighting the extent to which incomplete ground truth can obscure model performance. This suggests that LLM-based pipelines may serve not only as extraction tools but also as instruments for auditing and refining clinical registries, with direct implications for observational research where outcome misclassification can introduce systematic bias.

False-negative analysis revealed that missed events mostly stemmed from documentation gaps and prompt definition constraints rather than intrinsic model limitations, underscoring that sensitivity is bound by data completeness and prompt definition. The introduction of a ”suspicious” (SUSP) category allowed us to capture intermediate uncertainty. This surfaces cases with incomplete evidence or unmet confirmation criteria for targeted clinical review. In contrast to opaque, prior LLM-based classifiers [1, 9, 16, 27], our framework provides auditable evidence chains and enables linking output reasoning back to the initial evidence file. Structured error analysis identified failure modes such as note-date substitution and domain boundary ambiguities, notably, factual hallucinations were minimal, occurring in only two identified predictions. These insights into evidence-level error characterization are among the first reported for open-source LLMs applied to unstructured EHR data.

Our findings reinforce the view that unstructured EHR data contain critical clinical signals not fully captured in structured codes [14, 16, 27]. By transitioning from institution-specific, supervised models to a zero-shot architecture compatible with diverse clinical narratives, we significantly extend the utility of unstructured EHR data. Unlike previous studies restricted by input lengths [9, 16] or reliance on retrieval-augmented generation [28, 29], SCRIBE utilizes token-aware summarization to incorporate years of documentation within standard context windows. While temporal localization performance declines modestly with increasing initial record length, suggesting sensitivity to higher information density and redundancy in large longitudinal datasets, no abrupt performance degradation is observed even at extreme volumes. This indicates that our compression step effectively handles long-context inputs, supporting the feasibility of applying SCRIBE to large-scale clinical datasets. Notably, our pipeline achieves these results using a lightweight 4B-parameter model, suggesting that the explicit structuring of intermediate representations is a more critical determinant of performance than model scale alone.

Beyond oncology, this pipeline provides a generalizable framework for extracting patient-level outcomes without labeled training sets. However, these findings should be interpreted in the context of the following limitations. Evaluation depended on an imperfect reference standard, and generalizability across diverse health systems and non-English documentation requires further study. The transition to fully autonomous systems is hindered by the current necessity for human-in-the-loop clinical expertise. We opted for a lightweight, manual prompting approach rather than automated prompt-optimization suites such as DSPy [30], avoiding the need for labeled example generation, additional pipeline complexity, and computational overhead that such frameworks require. However, automated prompt optimization remains a promising avenue for future work, where users could optionally supply their own labeled examples or hand-crafted prompts to drive systematic prompt refinement.

Additionally, future work should focus on external validation across diverse institutions and the integration of multi-task label retrieval.

In summary, this study introduces a scalable, open-access pipeline that relies exclusively on text to generate precise, auditable patient labels. Demonstrated on lung cancer recurrence, this approach provides a foundation for constructing high-fidelity supervision signals from unstructured EHRs while also highlighting the potential of LLM-based systems to improve the completeness and reliability of real-world clinical data.

## 4 Methods

### 4.1 Study design and objective

We conducted a retrospective, multi-center observational study across eight hospitals within the Mass General Brigham (MGB) healthcare system. The primary objective was to develop and evaluate SCRIBE (Scalable Clinical Reasoning for Information and Biomarker Extraction), an open-source LLM-based pipeline for automated extraction of lung cancer recurrence events from longitudinal EHR [31] narratives. To preserve data privacy and ensure clinical portability, no model training or fine-tuning was performed; all experiments utilized frozen open-source models deployed exclusively behind the institutional firewall (on-premise).

### 4.2 Data sources and cohort construction

Unstructured clinical text was retrieved from the MGB Research Patient Data Registry (RPDR) [32–34], a centralized clinical data repository that aggregates longitudinal EHR from across the health system. RPDR consolidates clinical notes entered directly by clinicians into the EPIC EHR [35]. We selected two note types based on longitudinal coverage and clinical information density (Supplementary Table A1) [1]: ambulatory visit summaries (VIS) and inpatient progress reports (PRG). VIS are clinician-authored encounter summaries that capture the reason for the visit, history of present illness, review of systems, assessment, and management plan. PRG captures acute inpatient updates across clinical contexts, including perioperative, emergency department observation, and significant event documentation. Both modalities provide high patient-level coverage (96.2% for VIS; 97.5% for PRG) and dense documentation during a 90-day post-recurrence window (86.1% for VIS; 90.0% for PRG).

The study cohort was constructed by integrating data from three independent sources: i) the RPDR, which contains longitudinal clinical documentation including clinical notes, laboratory reports, and structured clinical data across the MGB health system; ii) the institutional sample of the Society of Thoracic Surgeons General Thoracic Surgery Database (STS GTSD) [36], capturing all lung resections performed at Massachusetts General Hospital; and iii) institutional cancer registries certified by the North American Association of Central Cancer Registries [37], capturing confirmed lung cancer diagnoses. The inclusion period was set to 2014–2024. Index surgery date was defined as the date of the first lung cancer resection or, when multiple resections occurred within 90 days, the date of the last. Of 4,646 patients with lung resections identified through the STS GTSD within the inclusion period, registry linkage followed by exclusions for incomplete longitudinal representation in the RPDR, advanced disease (stage III–IV) at surgery, invalid post-index diagnosis, and registry recurrence codes unsuitable for recurrence evaluation (70, 88, 99) [38] yielded a final registry-linked surgical cohort of 2,065 patients.

Fewer than 1% of patients were excluded because no clinical note was present on or after the recurrence date in the cancer registry, as their retention would have introduced structurally unavoidable false negatives independent of model performance. Approximately 61% of patients were female and 88–90% self-identified as White (Supplementary Table B2), consistent with regional lung cancer epidemiology. The overall recurrence-event prevalence was 10.12%, with a median follow-up of 6.86 years.

### 4.3 Ground truth cohorts

Two nested subsets were constructed using the registry-linked surgical cohort described in the preceding section to support pipeline development and progressive evaluation.

1. A development cohort (GT1, n = 37) [23, 39] had an enriched recurrence prevalence of 50% to ensure representation of the full spectrum of clinical recurrence presentations during iterative prompt development.
2. An ablation cohort consisted of 400 patients with an index surgery between 2015 and 2020 and at least two years of documented follow-up in the registry. After excluding 2 patients with no retrievable VIS or PRG, the effective ablation cohort comprises 398 patients (GT2), with a recurrence prevalence of 26.75%, consistent with estimates reported in the literature [40–43].

Of the 37 GT1 patients, 6 (16%) did not meet the inclusion criteria for GT2 and are therefore not represented in it. Registry-derived recurrence labels were withheld from the model throughout development.

### 4.4 Clinical event definition

Following semi-structured interviews with two board-certified thoracic radiologists (FJF, MK), we defined three longitudinal states to capture the trajectory of disease recurrence.

**Confirmed Recurrence (REC)** was defined as local lung cancer recurrence or a second primary lung cancer within the thorax. The diagnosis was supported by either tissue sampling, repeat lung surgery, or initiation of radiation therapy at least 6 months after the index surgery, consistent with established thresholds for separating residual disease from true recurrence [41, 44–48]. Second primary lung cancers were considered equivalent to recurrences for the purpose of this study. To account for both the periodicity of post-resection surveillance[48], the inherent documentation lag of the multi-step diagnostic cascade[49, 50] and the fact that a single lung cancer event generates multiple temporally dispersed administrative signals[8, 50, 51], diagnostic events occurring within a 3-month[1, 51, 52] window were consolidated into a single recurrence episode.

**Suspicious Event (SUSP)** captured clinically meaningful diagnostic escalation, such as indeterminate imaging findings or initiation of an invasive work-up, but without confirmation. This intermediate category, absent from standard registry definitions, serves a dual purpose: during global reasoning, it provides the model with a structured signal to distinguish transient radiological concern from true disease progression, enabling adjudication of whether suspicious imaging findings resolve or escalate into confirmed recurrence. For clinical reviewers, the SUSP label in the timeline provides a rapid, longitudinally ordered summary of diagnostic uncertainty across the full patient history, supporting the kind of trajectory-level judgment that isolated note review cannot.

**No Evidence of Recurrence (NOREC)** comprised stable imaging findings.

Detailed operational criteria, decision hierarchies, and adjudication rules are provided in Supplementary Methods E. These definitions were iteratively refined using false-positive and false-negative REC annotations on GT1, informed by clinical input, with no subsequent modifications upon transition to the ablation study on GT2.

### 4.5 Model selection and benchmarking

To assess whether lightweight open-source models, which can be run on local hospital premises, are capable of structured event extraction from medical free text, we implemented an auxiliary benchmark using Lung-RADS categories [53] extracted from radiology reports. As Lung-RADS categories are explicitly encoded in structured report sections, a deterministic regular-expression approach provided unambiguous ground-truth labels. We acknowledge that Lung-RADS extraction represents a more constrained task than longitudinal recurrence reasoning; this benchmark was accordingly used for feasibility screening and model selection rather than direct performance extrapolation. Eight open-source models ranging from 3.8B to 30B parameters were evaluated on schema adherence, hallucination rate, Lung-RADS classification accuracy, and inference throughput under matched on-premise hardware conditions (Supplementary Table B3). Each model output comprised two elements: a verbatim evidence string extracted from the source report and a derived Lung-RADS category. A model output was classified as hallucinated if the evidence string was not present verbatim in the source report, or if the Lung-RADS category derived from that evidence did not match the model’s asserted classification, capturing groundedness of the extraction relative to the source text rather than simple label agreement with the regex ground truth. Three models achieved the highest schema adherence (96.4%): Qwen3-4B-Instruct, llama-3.1–8B, and Qwen3-30B, while medgemma-4B and Qwen3-4B-Thinking showed complete schema non-compliance (0%). Hallucination rates similarly diverged: llama-3.1–8B exhibited the highest rate (25.5%), whereas Qwen3-4B-Instruct demonstrated the lowest rate (9.1%) among schema-compliant models. All schema-compliant models achieved perfect Lung-RADS classification accuracy against the regex-derived ground truth (100%). Qwen3-4B-Instruct further offered a favorable computational profile, with a mean throughput of 283.00 tokens per second and a mean VRAM consumption of 43.8 GB, balancing extraction reliability with on-premise deployment feasibility. All results can be found in Supplementary Table B3. Based on the highest schema adherence, the lowest hallucination rate among compliant models, and efficient inference characteristics, Qwen3-4B-Instruct-2507 was selected for all downstream experiments.

### 4.6 Design of the information extraction pipeline

Our SCRIBE pipeline employs a three-stage architecture designed to manage the density and redundancy of longitudinal clinical documentation. Step 1 - Note-level Extraction: Each patient note is processed independently to generate a structured JSON output containing the event label (REC/SUSP/NOREC), the event month, the model-derived reasoning chain, and the verbatim textual justification, to ensure auditability and traceability. Step 2 – Temporal Aggregation: Note-level predictions are aggregated at calendar-month resolution to consolidate overlapping clinical signals arising from repeated documentation of the same event across encounters. Proximate events within a 3-month window are merged into a single episode, consistent with the previously defined surveillance resolution. This deterministic grouping pre-structures chronologically ordered input for the downstream reasoning stage. When cumulative patient history exceeds the model’s context window, a token-budget-aware summarization step is applied, prioritizing months by clinical significance (REC > SUSP > NOREC) to preserve high-fidelity history for global reasoning. Stage 3 - Global Reasoning: The consolidated monthly timeline is processed by a judgment-stage LLM that performs global longitudinal reasoning to resolve cross-month contradictions and determine the final patient-level spatiotemporal recurrence trajectory.

Prompt engineering for both NLE and GR was performed iteratively using the GT1 cohort in collaboration with board-certified radiologists (FJF, MK). Clinical decisionmaking logic for identifying recurrence was first elicited through expert interviews and then translated into initial prompt formulations. We systematically identified model failure modes by comparing outputs against ground truth labels; these errors were then adjudicated with clinicians (FJF, JM) to distinguish between linguistic ambiguity and model reasoning constraints. This cycle was repeated until performance stabilized, the final prompts can be found here.

To assess sensitivity to prompt structure, alternative prompt variants were constructed using the prompting strategies CoT, ToT, schema-first prompting, and task sequencing, as well as combinations thereof, and Qwen3-4B-Instruct to reformulate the expert-derived base prompt. These variants were generated by reformulating the base prompt using Qwen3-4B-Instruct. A schema-modified variant replaced the multifield binary schema in the structured JSON output with a single multi-class output (Supplementary Methods D1). In addition, a few-shot prompt was constructed using representative failure cases identified during note-level prompt development.

For the global reasoning stage only six of the above defined eight prompt variants were compared. The output schema was fixed, as it was not used for downstream processing, and no few-shot examples were included due to the absence of radiologistadjudicated reference outputs at this stage.

All prompts, aggregation logic, and inference parameters were finalized on GT1 and held fixed for evaluation on the independent test cohort (GT2), with the exception of sampling temperature, which was varied in one dedicated experiment (Section 2.4).

### 4.7 Evaluation metrics

Performance was evaluated across two dimensions: **P**atient-level **R**ecurrence **D**etection (PRD), which assesses binary recurrence status, and **T**emporal **E**vent **L**ocalization (TEL), which evaluates recurrence event timing accuracy. For TEL, predicted recurrence events were classified as true positives if they fell within a tolerance window of the corresponding registry-recorded event. The empirically documented lag between true recurrence and its administrative or registry capture supports a window of 3 to 6 months as capturing the same underlying event [50, 52, 54]. Within this range, the optimal width was selected empirically by evaluating candidate values of ±3, ±4, ±5, and ±6 months on GT2. A window of ±4 months yielded the highest F1-score, and subsequent manual inspection by a trained analyst confirmed that this threshold produced the most coherent event clusters, with narrower windows creating artifactual splits of single recurrence episodes and wider windows merging distinct events. Optimal pairing of ground-truth and predicted events within this window was resolved using a minimum temporal distance matching algorithm (Supplementary 1). TEL precision, recall, and F1-score were computed based on these matched pairs and mean absolute error (MAE) in months was calculated over successfully matched true positive pairs. Additional temporal accuracy was reported at ±1, ±2, and ±3-month windows.

The expert adjudication of incorrectly classified patients and events followed a multi-step process. Determination of true recurrence relied on image interpretation by a board-certified thoracic radiologist, reflecting standard clinical practice. For false positive cases at both the patient and date level, a computer scientist first verified each case by locating the model’s verbatim evidence within the source record, with all findings independently confirmed by a trained analyst. Cases identified as clear model errors were not escalated further. Cases where the extracted evidence was clinically plausible and source documentation supported possible true recurrence were escalated to a board-certified thoracic radiologist (MK) for independent review of all available imaging. Where imaging was unavailable, the full medical history was reviewed to assess whether the prediction could be confirmed on clinical grounds. For false negative cases, a trained analyst reviewed the full patient history to identify any textual evidence of recurrence, after which the analyst and a computer scientist jointly assessed how the pipeline failed to detect it. No imaging review was performed for false negatives.

Adjudication was organized into three categories. Model errors were subdivided into temporal grounding errors, including note-date substitution, reference misplacement, and reasoning-step drift, and reasoning errors, comprising duplicate events, certainty conflation, and disconfirmatory evidence failure. Inherent study limitations captured misclassifications attributable to structural constraints rather than model failure, including domain knowledge boundaries, duplicate ascertainment, note-quality limitations, and ascertainment lag. Registry misclassifications denoted cases in which the model identified clinically plausible recurrences absent from or incorrectly recorded in the registry. All subcategories are detailed in Table 3.

### 4.8 Regex baseline

A deterministic rule-based baseline was developed in collaboration with thoracic radiologists following a top-down design strategy[55]. At the note level, patterns were organized into three families: strong recurrence indicators (e.g., explicit recurrence/progression terms, metastatic language, and interval size-increase phrases), uncertainty qualifiers (e.g., ’suspicious for,’ ’cannot rule out,’ ’worrisome’), and negative findings (e.g., ’no evidence of recurrence,’ ’stable disease,’ ’unchanged’) [56]. For each note, we computed family-specific scores as the count of matched patterns in each family and assigned the final label using rule-based comparisons among these three scores, with negative evidence capable of overriding positive signals when predominant. At the global reasoning stage, monthly event clusters were resolved by aggregating confidence scores across co-occurring notes, applying minimum confidence thresholds for REC (≤2) and SUSP (≤1) classification, and consolidating events within a 3-month temporal window by retaining the highest-confidence classification per cluster. Post-filtering logic and evaluation criteria were mirrored to those applied in SCRIBE to ensure a fair comparison.

### 4.9 Statistical analysis

Classification metrics (accuracy, F1-score, precision, recall) were compared across documentation modalities, prompt variations and pipeline stages using two-sided paired McNemar’s tests[57]; the exact variant was applied for comparisons with fewer than 25 discordant pairs, and the asymptotic variant with continuity correction was applied otherwise. Given non-normal distributions of temporal errors (Shapiro-Wilk *P* ≤ 0.001 for all strata[58]), MAE differences were evaluated using two-sided Wilcoxon signed-rank tests[59]. Confidence intervals (95%) were estimated via patient-level bootstrap resampling (B = 10,000). P-values were Bonferroni-adjusted to control the family-wise error rate across predefined comparison families $\left(\frac{\alpha }{m}\right)$ [60]. A two-sided *P* ≤ 0.05 was considered statistically significant after correction. Statistical analyses were performed using scipy (v1.10.1) and statsmodels (v0.13.5) in Python 3.10.

### 4.10 Ethics

The study was conducted with institutional review board approval and with a waiver of informed consent. All processing occurred within the institutional firewall. Outputs used for analysis were de-identified. Gender and race/ethnicity variables were obtained from the RPDR.

## Supplementary Material

1

See the Supplementary Information (PDF) for additional analyses.

Supplementary Files

This is a list of supplementary files associated with this preprint. Click to download.

- SCRIBESupplementary.pdf

## Figures and Tables

**Table 1:** Impact of global temporal reasoning on recurrence detection and event localization.

		PRD		TEL				
Modality	Stage	F1-score	Acc.	F1-score	Prec.	Recall	±1 mo	MAE
**PRG**	Note-level	70.9% (64.4–76.9)	80.5%	69.8% (62.8–76.7)	57.0%	89.9%	67.3%	1.29
	Global	72.9% (66.4–78.7)	82.3%	80.3% (75.3–85.5)	68.9%	96.3%	86.5%	0.63⋆⋆
**VIS**	Note-level	69.0% (62.2–75.2)	79.9%	71.6% (65.2–78.4)	60.1%	88.5%	69.6%	1.12
	Global	70.7% (64.0–76.8)	81.7%	77.9% (71.0–84.8)	70.3%	87.4%	86.7%	0.57⋆⋆
**VIS+PRG**	Note-level	69.3% (62.9–75.1)	78.0%	63.8% (57.7–70.3)	51.9%	82.6%	56.8%	1.61
	Global	70.9% (64.8–76.9)	79.3%	74.3% (68.2–80.7)	62.2%	92.2%	86.7%	0.52⋆⋆

PRD denotes patient-level recurrence detection; TEL denotes temporal event localization. Values in parentheses represent 95% CIs for F1 scores, MAE is reported in months.

⋆⋆ Denotes statistical significance (*P* < 0.025) for paired MAE differences between note-level and global stages, determined by Bonferronicorrected two-sided Wilcoxon signed-rank tests.

**Table 2:** Pairwise comparison of modality configurations in global reasoning.

Test	Wilcoxon W	MAE P-value	Δ MAE	PRD P-value	TEL P-value	PRD Test	TEL Test
PRG vs VIS	178.0	0.79	−0.06	0.79	0.00008⋆⋆	asymptotic	exact
PRG vs VIS + PRG	116.5	0.31	+0.11	0.35	0.63	asymptotic	exact
VIS vs VIS + PRG	177.5	0.37	+0.17	0.61	0.01⋆	asymptotic	asymptotic

Pairwise comparisons of modality configurations were conducted using two-sided Wilcoxon signed-rank tests (temporal) and McNemar’s tests (PRD and TEL).

Significance levels are indicated as follows:

⋆⋆ denotes Bonferroni-adjusted significance (*P* < 0.00167);

⋆ denotes nominal significance (*P* < 0.05) failing to survive Bonferroni correction.

Comparisons without notation did not reach statistical significance (*P* ≥ 0.05).

**Table 3:** Adjudicated reason categories across reviewed false-positive instances.

Category	Count (*n*)	Share of total (%)
Registry misclassification	64	47.76
Model failure	38	28.36
Study limitation	32	23.88

Distribution of adjudicated explanations assigned during error and discordance review. Percentages are reported relative to all reviewed instances (*n* = 134). Registry misclassification was the most common category, followed by model failure modes.

**Table 4:** Subcategory breakdown of adjudicated false-negative cases.

Model input	Main category	Subcategory	Count (*n*)	Evidence file modality	SUSP prediction
PRG	Study limitation	No available documentation	3	H&P=1	3/3
	Study limitation	Instruction boundary	3	PRG=3	2/3
	Model error	–	3	OP=2, PRG=1	3/3
VIS	Study limitation	No available documentation	13	OP=6, PRG=1, H&P=1	5/13
	Study limitation	Instruction boundary	3	PRG=2, OP=1	1/3
	Study limitation	Design choice	2	OP=1, PRG=1	0/2
	Model error	–	12	PRG=7, OP=4, H&P=1	8/12

Percentages are reported relative to all reviewed false-negative instances (*n* = 39). The final column summarizes the note types contributing to each subcategory.

## Data Availability

The MGB Dataset used in this study contains identifiable protected health information and cannot, therefore, be shared publicly. MGB investigators with appropriate IRB approval can contact the authors directly regarding data access.

## Code Availability

The code used to run SCRIBE and reproduce results can be found at https://github.com/FintelmannLabDevelopmentTeam/SCRIBE.git.
