# Supplementary material for: Longitudinal Language-Model Reasoning Enables Automated Labeling of Lung Cancer Recurrence from Unstructured Clinical Records
Source: Res Sq. 2026 May 21:rs.3.rs-9550278. Preprint. [Version 1] doi: 10.21203/rs.3.rs-9550278/v1 (PMC13229083; doi:10.21203/rs.3.rs-9550278/v1)
Supplement: 1 [file NIHPPRS9550278V1-supplement-1.pdf]

Supplementary Material

PRD denotes patient-level recurrence detection; TEL denotes temporal event localization. Values are reported as percentages unless otherwise indicated. 95% confidence intervals are calculated for PRD accuracy, PRD F1, and TEL F1. Classification differences were evaluated using paired McNemar tests for PRD and TEL. Temporal differences were evaluated using paired Wilcoxon signed-rank tests on MAE. All quantitative experiments were conducted using the ablation cohort (GT2, n=398) unless otherwise stated.

Appendix A Text Modalities

Table A1: **Characteristics and longitudinal availability of unstructured EHR modalities.** Per modality, we report patient-level coverage within the study cohort, median longitudinal coverage (days between first and last note per patient), and note availability within ±90 days of confirmed recurrence among REC patients. Coverage metrics were calculated after application of inclusion and exclusion criteria.

Code	File	Description / Definition	Patient coverage	Period coverage (days, median)	REC window coverage (90 days)
**VIS**	Visit Summary (Ambulatory Notes)	Clinician-authored encounter summaries including reason for visit, history of present illness, review of systems, vitals, assessment and plan, care instructions, and medication reconciliation.	96.20%	2675	86.12%
**PRG**	Progress Reports	Inpatient progress documentation capturing interval clinical updates, including perioperative, emergency, nursing, or specialty-specific notes.	97.51%	2702.5	89.95%
PUL	Pulmonary Reports	Specialty reports containing pulmonary function test interpretations and impressions, including spirometry, lung volumes, and walk tests.	20.97%	0	11.00%
DIS	Discharge Summary	End-of-hospitalization summaries documenting admission and discharge dates, hospital course, patient status, follow-up plans, and medication lists. Content may be partially templated.	96.54%	290.5	55.02%
RAD	Radiology Notes	Narrative imaging reports structured into technique, comparison, findings, and impression sections for CT, PET, MRI, and other imaging studies.	99.46%	2637	97.13%
LNO	Longitudinal Notes	Legacy ambulatory notes authored in the Longitudinal Medical Record (LMR) system. Content varies and may include inpatient or outpatient documentation prior to EPIC transition.	63.33%	346	11.48%
HNP	History of Present Illness	Structured longitudinal health maintenance data including immunizations, vital signs, physical findings, and related preventive care information.	91.18%	520	61.72%
OPN	Operative Notes	Narrative procedural documentation including indications, intraoperative findings, specimens, blood loss, condition at end of procedure, and follow-up plans.	96.98%	573	72.25%
END	Endoscopy	Procedure reports documenting indications, findings, impression, and recommendations from endoscopic examinations.	42.27%	454	3.83%
PAT	Pathology Reports	Diagnostic reports including specimen description, adequacy, histopathologic interpretation, and final diagnosis.	97.46%	1231	80.38%

Appendix B Dataset Characteristics

Table B2: Cohort demographics across ground truth datasets.

Characteristic	GT1 ($n = 38$)	GT2 ($n = 398$)	surgical cohort ($n = 2{,}065$)
Age (years)			
Mean (SD)	75.97 (8.25)	74.32 (9.27)	73.68 (9.84)
Median [Range]	75.5 [56–93]	75.0 [34–98]	74.0 [23–99]
Age groups (%)			
30–55	0.0%	3.25%	3.83%
55–70	18.42%	24.50%	25.71%
≥ 70	81.58%	72.25%	70.31%
Sex (%)			
Female	63.16%	61.75%	61.36%
Male	36.84%	38.25%	38.64%
Race / Ethnicity (%)			
White	89.47%	91.25%	88.47%
Black	5.26%	1.75%	2.91%
Asian	0.00%	2.75%	3.87%
Other / Unknown	5.26%	4.25%	4.75%
Recurrence prevalence	50.0%	26.75%	12.2%

Model Benchmarking

The evaluated models were Llama-3.1-8B-Instruct (Meta, 8B parameters) [61], an instruction-tuned general-purpose model with strong structured output compliance; Mistral-NeMo-Instruct-2407 (Mistral AI, 12B parameters) [62], a multilingual model developed in collaboration with NVIDIA featuring a 128k token context window and quantization-aware training; Phi-4-mini-Instruct (Microsoft, 3.8B parameters) [63], a compact model trained on reasoning-rich synthetic data representing the smallest memory footprint in the candidate set; MedGemma-4B-IT (Google, 4B parameters) [64], a medically specialized vision-language model built on Gemma 3 4B and fine-tuned on medical image-text pairs, evaluated in text-only mode; and four members of the Qwen3 family (Alibaba Cloud) [65], specifically Qwen3-4B-Instruct-2507 (4B), Qwen3-4B-Thinking-2507 (4B), Qwen3-8B (8B), and Qwen3-30B-A3B-Instruct-2507 (30B).

Table B3: **Open-source language model benchmark on Lung-RADS category extraction from radiology reports.**
Schema adherence reflects the proportion of model outputs conforming to the required JSON output structure. Hallucination rate reflects the proportion of outputs asserting categorical values absent from the source text. Lung-RADS accuracy reflects agreement with deterministic regular-expression-derived ground-truth labels. Latency and throughput were measured under matched on-premise hardware conditions. The selected model (Qwen3-4B-Instruct) is shown in bold.

Model	Params (B)	Schema adherence (%)	Hallucination rate (%)	Lung-RADS accuracy (%)	Mean latency (sec)	Mean tokens / sec	GPU memory used (MB)
llama-3.1-8b-instruct	8	96.36	25.45	100	1.85	395.20	44594.65
medgemma-4b-it	4	0	0	N/A	278.96	9.16	1
mistral-nemo-instruct-2407	12	96.36	30.91	100	2.21	318.63	44455.02
Phi-4-mini-Instruct	3.8	0	45.45	N/A	2.85	242.47	45162.36
**Qwen3-4B-Instruct-2507**	**4**	**96.36**	**9.09**	**100**	**2.57**	**283.00**	**44697.06**
Qwen3-4B-Thinking-2507	4	0	25.45	N/A	9.16	104.45	45423.83
Qwen3-8B	8	0	3.64	N/A	10.52	90.52	43886.57
Qwen3-30B-A3B-Instruct-2507	30	96.36	14.55	100	7.44	96.32	44929.84

Appendix C Pipeline Evaluation

Table C4: **REGEX classification performance by modality and reasoning stage.**

		PRD		TEL			
Modality	Stage	Accuracy (95% CI)	F1 (95% CI)	Precision	Recall	F1 (95% CI)	MAE (months)
VIS	Note-level	38.3% (33.6–43.2)	43.9% (38.0–49.7)	21.9%	72.1%	33.6% (29.5–37.9)	1.09
	Global Reasoning	35.8% (31.2–40.6)	43.5% (37.6–49.0)	17.0%	51.3%	25.6% (21.0–30.3)	1.90
PRG	Note-level	33.2% (28.8–38.0)	43.3% (37.4–48.8)	19.3%	75.0%	30.7% (27.3–34.6)	1.25
	Global Reasoning	33.2% (28.8–38.0)	43.0% (37.2–48.5)	23.9%	63.5%	34.7% (30.0–39.7)	1.40

C.1 Statistical evaluation per modality

Table C5: **Pairwise statistical comparison of NLE vs. GR per modality.** Statistical significance was defined using a Bonferroni-adjusted threshold of $\alpha = 0.0167$ to account for 3 pairwise comparisons. $\star\star$ Denotes significance at the adjusted level ($P < 0.0167$), $\star$ Indicates nominal significance ($P < 0.05$) that did not survive Bonferroni correction.

Test	Δ MAE	Wilcoxon W	MAE P-value	PRD P-value	TEL P-value
PRG	0.7	536.5	$0.0001^{\star\star}$	0.07	$0.002^{\star\star}$
VIS	0.58	196.0	$0.001^{\star\star}$	0.21	1.0
VIS+PRG	1.09	322.0	$4.13\text{e-}07^{\star\star}$	0.06	$0.004\star\star$

C.2 Inference repeatability

Table C6: **Pairwise statistical comparison of repeated runs (global reasoning, PRG)** No comparison reached statistical significance.

Test	Wilcoxon W	MAE P-value	ΔMAE	PRD P-value	TEL P-value
Run 1 vs Run 2	40.0	0.696	−0.06	0.608	0.267
Run 1 vs Run 3	109.5	0.084	+0.05	0.207	0.185
Run 2 vs Run 3	6.5	0.098	+0.11	0.774	1.000

Table C7: **Run-to-run repeatability across three independent executions (PRG)**

		**PRD**		**TEL**		
	Stage	Accuracy (95% CI)	F1 (95% CI)	Precision	Recall	F1 (95% CI)
Run 1	Note-level	80.5% (76.3–84.1)	70.9% (65.1–76.9)	57.5%	89.9%	69.8% (62.8–76.7))
	Global reasoning	82.1% (78.3–85.8)	72.9% (66.4–78.7)	68.9%	96.3%	80.3% (75.3–85.5)
Run 2	Note-level	79.9% (75.7–83.5)	69.0% (62.2–75.2)	60.1%	88.5%	71.6% (65.2–78.4)
	Global reasoning	81.7% (77.6–85.2)	70.7% (64.0–76.8)	70.3%	87.4%	77.9% (71.0–84.8)
Run 3	Note-level	80.9% (76.8–84.5)	70.1% (63.3–76.2)	59.9%	90.4%	72.0% ([66.2–78.3)
	Global reasoning	82.2% (78.1–85.6)	70.8% (64.0–77.0)	69.2%	92.0%	79.0% (73.0–85.2)

C.3 Inference model-temperature impact

Table C8: **Classification performance across sampling temperatures (global reasoning, PRG).**

		PRD		TEL		
Temp	Stage	PRD Accuracy (95% CI)	PRD F1 (95% CI)	TEL Precision	TEL Recall	TEL F1 (95% CI)
1.0	Note-level	79.9% (75.7–83.5)	69.2% (62.5–75.3)	66.4%	92.4%	77.3% (71.1–83.5)
	Global Reasoning	82.9% (78.9–86.3)	72.6% (66.1–78.6)	67.9%	86.7%	76.2% (69.3–82.9)
0.7	Note-level	78.1% (73.8–81.9)	66.4% (59.4–72.7)	60.6%	94.0%	73.7% (66.9–80.7)
	Global Reasoning	79.9% (75.4–83.3)	68.3% (75.7–83.5)	69.5%	91.0%	78.8% (72.7–84.8)
0.1	Note-level	80.5% (76.3–84.1)	70.9% (65.1–76.9)	57.5%	89.9%	69.8% (62.8–76.7)
	Global Reasoning	82.3% (78.3–85.8)	72.9% (66.4–78.7)	68.9%	96.3%	80.3% (75.3–85.5)

Table C9: **Pairwise statistical comparison of sampling temperatures (global reasoning, PRG).** Statistical significance was defined using a Bonferroni-adjusted threshold of $\alpha = 0.0167$ to account for 3 pairwise comparisons. ⋆⋆ Denotes significance at the adjusted level ($P < 0.0167$), ⋆ Indicates nominal significance ($P < 0.05$) that did not survive Bonferroni correction.

Test	Δ MAE	Wilcoxon W	MAE P-value	PRD P-value	TEL P-value
Temp 1.0 vs 0.7	-0.01	79.0	0.316	0.026⋆	1.0
Temp 1.0 vs 0.1	-0.06	167.0	0.822	0.35	0.011⋆⋆
Temp 0.7 vs 0.1	-0.05	148.0	0.316	0.609	0.003⋆⋆

C.4 Inference NLE-prompt impact

Table C10: **Note-level extraction performance across prompt variants (PRG).**

Prompt	PRD		TEL		
	Accuracy (95% CI)	F1 (95% CI)	Precision	Recall	F1 (95% CI)
Base	80.5% (76.3–84.1)	70.9% (65.1–76.9)	57.7%	89.9%	69.8% (62.8–76.7)
CoT	79.4% (75.2–83.1)	67.2% (60.2–73.6)	58.1%	90.9%	70.9% (63.4–78.7)
ToT	72.9% (68.3–77.0)	57.1% (49.4–64.2)	43.2%	82.4%	56.7% (50.0–64.2)
Few-shot	80.9% (76.8–84.5)	69.8% (62.9–76.0)	63.8%	92.2%	75.4% (69.4–81.4)
Multi-class JSON	78.6% (74.4–82.4)	63.2% (55.6–70.1)	54.2%	89.7%	67.5% (60.6–75.2)
Schema-First	73.6% (69.0–77.6)	59.8% (52.4–66.7)	48.1%	83.5%	61.0% (54.3–68.5)
Task Sequencing	71.6% (67.0–75.8)	59.2% (52.1–65.9)	46.5%	81.4%	59.2% (52.8–65.6)
Merge Techniques	74.9% (70.4–78.9)	62.7% (56.5–69.9)	46.7%	79.8%	59.0% (52.4–65.9)

Table C11: **Pairwise statistical comparison across note-level prompt variants (PRG).** Significance thresholds were adjusted for seven pairwise comparisons against the manually optimized base configuration. ⋆⋆ Denotes statistical significance after Bonferroni correction ($\alpha = 0.0071$); ⋆ indicates nominal significance ($P < 0.05$) that did not survive correction. Values without notation are non-significant ($P \geq 0.05$).

Comparison	PRD P-value	TEL P-value
Base vs CoT	0.665	0.281
Base vs ToT	0.001⋆⋆	< 0.001 ⋆⋆
Base vs Few-shot	0.892	0.856
Base vs Multi-class JSON	0.532	0.001⋆⋆
Base vs Schema-First	0.004⋆⋆	0.002⋆⋆
Base vs Task Sequencing	< 0.001 ⋆⋆	0.004⋆⋆
Base vs Merge Techniques	0.013⋆	0.001⋆⋆

C.5 Inference GR-prompt impact

Table C12: **Global reasoning performance across prompt variants (PRG).**

Prompt	PRD		TEL		
	Accuracy (95% CI)	F1 (95% CI)	Precision	Recall	F1 (95% CI)
Base	82.3% (78.0–85.5)	72.9% (66.4–78.7)	68.9%	96.3%	80.3% (75.3–85.5)
CoT	80.6% (76.4–84.2)	70.5% (63.8–76.4)	63.5%	93.4%	75.6% (69.7–81.7)
ToT	79.3% (75.0–83.0)	69.4% (62.8–75.4)	60.7%	92.5%	73.3% (67.1–79.5)
Task Sequencing	80.8% (76.6–84.4)	71.0% (64.4–76.8)	63.5%	94.4%	75.9% (70.1–82.0)
Schema-First	81.6% (77.4–85.1)	72.0% (65.6–77.9)	66.0%	89.8%	76.1% (70.4–82.0)
Merge Techniques	81.3% (77.2–84.8)	71.8% (65.3–77.6)	66.9%	93.5%	78.0% (72.2–83.9)

Table C13: **Pairwise statistical comparison across global reasoning prompt variants (PRG).** Statistical significance was defined using a Bonferroni-adjusted threshold of $\alpha = 0.01$ to account for five comparisons against the base configuration. $\star\star$ Denotes significance at the adjusted level ($P < 0.01$); $\star$ indicates nominal significance ($P < 0.05$) that did not survive Bonferroni correction.

Comparison	PRD *P*-value	TEL *P*-value
Base vs CoT	0.549	0.289
Base vs ToT	0.031 $\star$	0.180
Base vs Task Sequencing	1.000	0.070
Base vs Schema-First	0.344	0.375
Base vs Merge Techniques	0.219	0.375

C.6 Expert adjudication and error analysis

Table C14: **Subcategory breakdown of adjudicated reason assignments among false-positive cases.** Detailed categorization of reviewed false-positive instances across registry misclassifications, model errors, and study limitations. Percentages are reported relative to all reviewed false-positive instances ($n = 134$).

Main category	Subcategory	Count (n)	Share of total (%)
Model error	Temporal grounding – note date substitution	16	11.94
	Temporal grounding – reasoning step drift	8	5.97
	Temporal grounding – reference misplacement	5	3.73
	Reasoning error – duplicate event	5	3.73
	Reasoning error – certainty conflation	2	1.49
	Reasoning error – disconfirmatory evidence failure	2	1.49
Registry misclassification	True positive	44	32.84
	Missing imaging; text true positive	19	14.18
	True positive (date correction)	1	0.75
Study limitation	Domain knowledge boundary	17	12.69
	Duplicate ascertainment	12	8.96
	Note-quality limitation – typographic error	2	1.49
	Ascertainment lag	1	0.75

Table C15: **Taxonomy of adjudicated categories and underlying failure mechanisms.** Subcategories are mapped to their corresponding error mechanisms. Model errors primarily reflect temporal grounding and reasoning failures, whereas registry misclassifications and study limitations arise from imperfections in structured labels, incomplete source material, or annotation constraints rather than incorrect model inference alone.

Main category	Subcategory	Failure mode description
Model error	Temporal grounding – note date substitution	Event was linked to the note date rather than the clinically relevant event date, often in copy-forward documentation
	Temporal grounding – reasoning step drift	Correct temporal information was extracted on the note level, but the final reasoning step reassigned the event to an incorrect date
	Temporal grounding – reference misplacement	A real date from the record was attached to the wrong clinical event or episode (attribute-binding failure)
	Reasoning error – duplicate event	Previously identified recurrence evidence was incorrectly treated as new recurrence rather than part of the same episode
	Reasoning error – certainty conflation	Suspicious or provisional evidence was incorrectly labeled as confirmed recurrence despite correct finding and timing
	Reasoning error – disconfirmatory evidence failure	Negated evidence in the record was not incorporated, resulting in an unsupported positive classification
Registry misclassification	True positive	Model prediction was clinically correct, but the reference registry label was incorrect
	Missing imaging; text true positive	Model prediction was clinically correct, registry label was incorrect, CT evidence not available, validation through clinical history
	True positive (date correction)	Model predicted a correct recurrence absent from the registry, but with a slight temporal difference to the true event
Study limitation	Domain knowledge boundary	Disease classification falls outside the task scope or field of view, preventing unambiguous recurrence assignment; classification was clinically defensible under ambiguity
	Duplicate ascertainment	A single unresolved recurrence episode was counted multiple times because repeated triggers each satisfied the annotation criteria
	Note-quality limitation – typographic error	Errors in the source note, such as copied-forward or mistyped dates, propagated into model output despite otherwise reasonable interpretation
	Annotation boundary	Annotation criteria requires confirmatory clinical action, which occasionally postdates the earliest documented suspicion. Where documentation reflects only clinical suspicion or deferred action due to patient circumstances, the pipeline correctly assigns SUSP rather than REC, diverging from registry REC labels.
	No available documentation	Recurrence is documented in the record but absent from the selected unstructured text modality.
	Design choice	The pipeline anchors to the earliest confirmatory event which precedes the registry records', when the difference exceeds the matching window the prediction goes unmatched.

Table C16: **Performance comparison: registry-corrected versus unadjusted ground truth (VIS and PRG).** Bonferroni correction was not applied; a single pairwise comparison was performed per stage and modality. ⋆Statistically significant PRD improvement with corrected ground truth. TEL metrics were unchanged across all comparisons (no discordant pairs), confirming that temporal localization performance was unaffected by label correction.

Mod	Stage	GT	PRD Accuracy (95% CI)	PRD F1 (95% CI)	TEL Precision	TEL Recall	TEL F1 (95% CI)	PRD *P*-value	TEL *P*-value
VIS	Note-level	Unadjusted	79.9% (75.7–83.5)	69.0% (63.9–76.5)	60.1%	88.5%	71.6% (65.2–78.4)	Ref	Ref
		Adjusted	81.7% (77.6–85.2)	72.5% (66.1–78.3)	64.4%	88.9%	71.6% (68.8–81.2)	0.016⋆	1.000
	Global Reasoning	Unadjusted	81.9% (77.8–85.4)	70.7% (64.0–76.8)	70.3%	87.4%	77.9% (71.0–84.8)	Ref	Ref
		Adjusted	86.9% (83.3–89.9)	81.3% (76.0–86.0)	77.6%	88.6%	82.8% (78.1–87.1)	< 0.001⋆	1.000
PRG	Note-level	Unadjusted	80.5% (76.3–84.1)	70.9% (65.1–76.9)	57.5%	89.9%	69.8% (62.8–76.7)	Ref	Ref
		Adjusted	82.3% (78.2–85.7)	74.3% (67.1–79.0)	61.1%	89.7%	72.7% (66.5–79.1)	0.016⋆	1.000
	Global Reasoning	Unadjusted	82.3% (78.3–85.8)	72.9% (66.4–78.7)	68.9%	96.3%	80.3% (75.3–85.5)	Ref	Ref
		Adjusted	88.9% (85.4–91.6)	84.6% (79.8–88.9)	76.4%	93.6%	84.1% ([80.0–88.2)	< 0.001⋆	1.000

C.7 Inference demographic bias

Table C17: **Subgroup performance evaluation by sex, race, and age (global reasoning, PRG).** Power estimates reflect post-hoc analysis for detecting a 10 percentage-point difference in TEL F1 relative to the overall cohort reference value at $\alpha = 0.05$ and 80% power. Minimum detectable effect (MDE) at 80% power is shown for each subgroup. Racial minority subgroups were substantially underpowered, with MDEs ranging from 25 to 32 percentage points; the absence of a detected performance disparity should not be interpreted as evidence of equitable performance across populations.

Subgroup	N	PRD Sens.	PRD Spec.	TEL F1	TEL MAE	Power (10pp)	MDE 80%
Overall	398	91.8%	79.0%	78.1%	0.62	—	—
Female	246	92.2%	78.0%	77.2%	0.64	0.57	13.0pp
Male	152	90.9%	80.5%	80.5%	0.58	0.57	12.8pp
White / Caucasian	363	90.6%	79.3%	76.9%	0.55	0.24	21.7pp
Black / African American	7	100.0%	60.0%	100.0%	1.50	0.09	32.4pp
Asian	11	100.0%	85.7%	80.0%	1.00	0.11	29.2pp
Other / Unknown	17	100.0%	75.0%	88.9%	1.00	0.13	26.1pp
Age 30–55	13	100.0%	90.0%	75.0%	0.67	0.12	27.8pp
Age 55–70	98	95.0%	74.4%	74.5%	0.53	0.48	14.2pp
Age $\geq$ 70	287	90.8%	80.3%	79.1%	0.64	0.51	14.0pp

Appendix D Methods

D.1 Evaluation: PRD & TEL

Algorithm 1: Hierarchical Recurrence Evaluation and Clustering

Input: Ground Truth $\mathcal{G}$, Predictions $\mathcal{P}$, Threshold $\tau = 3$ months
Output: PRD and TEL
Filter $\mathcal{P}$ by $\mathcal{G}_{\text{EMPI}}$ and remove events $\leq T_{\text{early-post-surgery}}$;
Censor $\mathcal{P}$ beyond date of last registry contact t_{last};
`// Temporal Sliding-Window Clustering`
Function `Cluster`(*events*, τ)**:**
  Sort events by month;
  Group events into cluster C if $\Delta t \leq \tau$;
  $L_C \leftarrow$ hierarchical: REC $\succ$ SUSP $\succ$ NOREC $\in C$;
  $t_C \leftarrow \text{median}(t \in C)$;
  return $\{C_1, C_2, ...C_n\}$;
`// TEL: Minimum Distance Assignment`
foreach *TP Patient* **do**
  $\mathcal{C}_{GT} \leftarrow$ `Cluster`$(\mathcal{G}_P, \tau)$;
  $\mathcal{C}_{pred} \leftarrow$ `Cluster`$(\mathcal{P}_P, \tau)$;
  Perform greedy matching based on $\min|t_{\mathcal{C}_{GT}} - t_{\mathcal{C}_{pred}}|$;
  Classify unmatched $\mathcal{C}_{pred}$ as FP_{Stage2} if distance $>$ Tolerance;
end

D.2 NLE output schema

Fig. D1: Structured JSON output schemas for the multi-class and binary Note-Level Extraction.

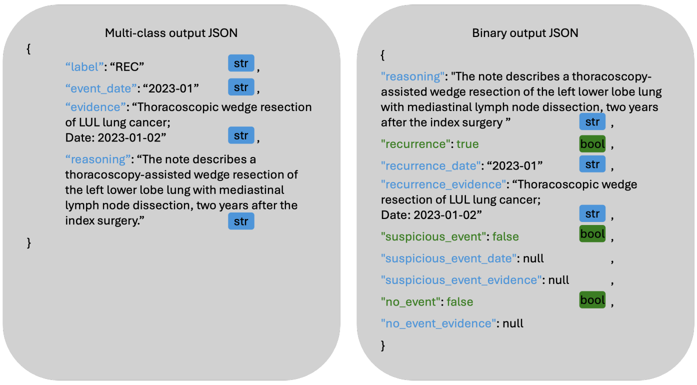

Appendix E Operational Criteria, Decision Hierarchies, and Adjudication Rules

This section details the formal logic and operational definitions used for the automated extraction and longitudinal adjudication of lung cancer recurrence (REC), suspicious events (SUSP), and no evidence of recurrence (NOREC).

E.1 Note-level extraction criteria

Individual clinical documents were classified based on five specific recurrence triggers ($R_1 - R_5$) and three suspicious event triggers ($S_1 - S_3$).

E.1.1 Confirmed REC triggers

A note is labeled as REC if at least one of the following is documented after the index surgery:

- **R1 (Definite PET):** PET imaging explicitly stating "definite" or "confirmed" recurrence.
- **R2 (Positive pathology):** Malignant biopsy (e.g., EBUS, CT-guided) or pathology report from lung tissue or hilar/mediastinal nodes.
- **R3 (Repeat surgery):** Subsequent lung resection (e.g., wedge, lobectomy) performed >2 months post-index surgery.
- **R4 (Explicit diagnosis):** Clinical statements of "recurrent lung cancer" or "metachronous primary".
- **R5 (Delivered Therapy):** Initiation of SBRT, new radiation, or systemic therapy (chemotherapy/immunotherapy) directed at a lung-specific lesion.

E.1.2 Suspicious event (SUSP) triggers

A note is labeled as SUSP if it describes clinical escalation without meeting REC gates:

- **S1 (Non-positive invasive workup):** Biopsies or pathology with "benign," "atypical," or "indeterminate" results.
- **S2 (Indeterminate PET):** FDG-avid findings described as "concerning," "favored," or "cannot exclude malignancy" without "definite" language.
- **S3 (Concrete escalation planning):** Explicit orders or scheduling for PET, biopsy, or surgery to evaluate a new finding (excluding routine surveillance CT).

E.2 Decision hierarchies and hard constraints

To ensure specificity and handle the inherent noise of clinical documentation, the following hierarchy and constraints were applied:

- **CT-only rule:** Radiological findings alone (e.g., "enlarging nodule," "highly suspicious CT") **never** trigger REC or SUSP. CT findings are classified as NOREC unless followed by PET, biopsy, or treatment.

- **History vs. event:** Phrases such as "status post (s/p)" or "history of" do not trigger events unless they describe diagnostic/treatment actions occurring within the current clinical timeframe (within the year of the current note).
- **Anatomic Specificity:** REC is restricted to the thorax (lung regions or mediastinal nodes). Isolated distant metastases (e.g., brain, liver) without lung involvement are excluded from the REC label.
- **Temporal Buffers:** Any event occurring $\leq$2 months after the index surgery is treated as a synchronous primary or residual disease (NOREC).

E.3 Global adjudication and reasoning

The global reasoning layer consolidates note-level extractions into a patient-level timeline using a three-phase workflow:

Mandatory workflow

Phase A1 (Global Read): All episodes and their supporting evidence are reviewed in full before any label is assigned, allowing formation of a coherent patient course across time.

Phase A2 (Event Ledger): All potential events are extracted into an internal ledger. The model must resolve relative time markers (e.g., "recent biopsy") to specific dated anchors. Any event that clearly refers to an already-anchored confirmed episode is marked HISTORY_ONLY and is prohibited from generating a new episode.

Phase B (Episode Adjudication): Episodes are validated and repaired as follows:

- **Upgrade or downgrade**: An episode is upgraded to REC if a confirmation gate (see E.1.1) is met; downgraded to SUSP or NOEVENT if it is not.
- **Split**: A NOEVENT episode containing an escalation action within its interval is split at the earliest escalation month supported by evidence.
- **Merge**: Episodes describing the same lesion, workup chain, or treatment course are merged; the highest escalation month is retained as anchor.
